# Supplementary material for: Direct visualization of MCM helicase activation and replisome coupling in situ
Source: bioRxiv. 2026 Aug 24:2026.08.21.746258. Preprint. [Version 1] doi: 10.64898/2026.08.21.746258 (PMC13536934; doi:10.64898/2026.08.21.746258)
Supplement: Supplementary file 1 [file NIHPP2026.08.21.746258v1-supplement-1.pdf]

bioRxiv preprint doi: https://doi.org/10.64898/2026.08.21.746258; this version posted August 24, 2026. The copyright holder for this preprint (which was not certified by peer review) is the author/funder, who has granted bioRxiv a license to display the preprint in perpetuity. It is made available under a CC-BY 4.0 International license.

Supplementary Note 1

To estimate the active SH form, we calculated the enrichment of the MINFLUX nearest-neighbor (NN) distribution over the simulated monomer and dimer distributions per ROI. In S phase, an enrichment within the 30–85 nm window was observed. Identifying this population as the SH form is supported by the fact that all measurements of G1-phase cells showed no significant enrichment (MCM2: 3 ± 2%; MCM2 with siRAD21 depletion: 0 ± 2%; MCM4: 2 ± 2%) (Fig. 2h, 2j, Extended Fig. 8f). Additionally, after activating dormant origins (ATRi+0.2 mM HU) we observed a more distinct peak in the NN distance and an increase in enrichment within the 30–85 nm range (Fig. 4c). Lastly, MINFLUX measurements of MCM4 also exhibited a distinct peak in this same region (Fig. 3f). Therefore, we used the enriched NN fraction as an estimate for the SH fraction. The mean enrichment across MCM foci during S phase was 8.4 ± 1.3% for MCM2, 9 ± 3% for MCM4, and 10.8 ± 1.7% for MCM2 with ATRi+0.2 mM HU. %) (Fig. 2h, 2j and Extended Fig. 6f). No corrections were performed for 3D orientation or labeling fraction. Note that the estimates are only snapshots of active MCM at a given time, thus will bias it towards it being a lower estimate. All values are presented as mean ± SEM.
